# Relationship between perceived social support and disability with the mediating role of perceived stress among older adults

**DOI:** 10.1186/s12877-024-04871-z

**Published:** 2024-03-20

**Authors:** Leila Dehghankar, Saman Valinezhad, Mohammad Amerzadeh, Farnoosh Zarabadi Poor, Zahra Hosseinkhani, Seyedeh Ameneh Motalebi

**Affiliations:** 1 Social Determinants of Health Research Center, Research Institute for Prevention of Non-Communicable Diseases, Qazvin University of Medical Sciences, Qazvin, Iran; 2 Student Research Committee, Qazvin University of Medical Sciences, Qazvin, Iran; 3 Metabolic Diseases Research Center, Research Institute for Prevention of Non-Communicable Diseases, Qazvin University of Medical Sciences, Qazvin, Iran

**Keywords:** Social support, Disability, Stress, Psychological, Aged

## Abstract

**Background:**

Social support is essential for individuals to cope with difficult circumstances. Older adults with disabilities face significant challenges in engaging with everyday activities. The current study examines the mediating role of perceived stress in the association between perceived social support and disability among Iranian community-dwelling older adults.

**Methods:**

In this descriptive and cross-sectional study, 300 older adults were selected using cluster sampling from January to June 2022. The data were collected using the Multidimensional Scale of Perceived Social Support (MSPSS), the World Health Organization Disability Assessment Schedule 2.0 (WHODAS 2), and the Cohen Perceived Stress Scale-14 (PSS-14). The collected data was analyzed using structural equation modeling (SEM) in Mplus.

**Results:**

The mean age of older adults was 68.71 ± 6.13 years, ranging from 60 to 85 years old. The results of this study revealed a significant relationship between perceived social support and disability (β=-0.20, SE = 0.06, *p* < 0.001) and perceived stress and disability (β = 0.50, SE = 0.05, *p* < 0.001). The results also confirmed the mediating role of perceived stress in the relationship between perceived social support and disability (β=-0.17, SE = 0.03, *p* < 0.001).

**Conclusion:**

The results indicated that increasing social support could reduce disability by decreasing perceived stress. These results have important implications for policymakers and healthcare professionals in promoting healthy aging.

## Background

Population aging is anticipated to lead to significant increases in the number of persons with chronic diseases, disabilities, and functional limitations [1]. Based on the International Classification of Functioning, Disability, and Health (ICF) definition, disability is an umbrella term for impairments, activity limitations, and participation restrictions [2, 3]. The prevalence of chronic disability conditions is higher among older adults, significantly affecting their ability to engage in daily activities including social interactions. Long-term social consequences arise from these conditions and making older adults highly vulnerable to psychological and social stressors [4–6].

Stress is a common risk factor for physical and psychological health issues among older adults. Perceived stress is the set of emotions or thoughts that an individual experience in response to a particular stressor at a given time [7, 8]. Older adults experience higher levels of perceived stress than other age groups. Raisvandi et al. (2023) reported a high level of stress (40.2%) among a sample of Iranian older adults [9]. Older adults who experience high levels of stress are at risk for a variety of negative health outcomes, including cardiovascular disease, immune system dysfunction, sleep disturbances, and depression [10]. Stress-inducing events may require various sources and types of support [11]. As Yang (2006) showed that stress increased perceived social support, and disability, as a stress-inducing factor, leads to increased in instrumental social support [12].

Perceived social support refers to the perception of caring for others and the benefit of a trusted network to turn in times of need and during critical and challenging moments [13]. Social support is crucial for the well-being of older adults as it provides emotional reinforcement, improves health-promoting behaviors, and facilitates friendly relationships [14, 15]. As older adults experience declines in their physical, cognitive, and mental abilities, they require more social support [16].

Older adults who actively engage in social networks and receive informal social support tend to have more positive mental and physical health compared to those who experience social isolation [17]. Perceived social support is a crucial determinant of life satisfaction, quality of life, and adaptation to stressful conditions in older adults [18]. According to the buffer-stress hypothesis, perceived social support can protect individuals against adverse impacts of high stress [19]. This suggests that having a high level of social support can moderate the negative impacts of stress on various aspects of health and well-being [20]. Social support can help individuals cope with stress and lead to fewer psychological and physical symptoms and illnesses by providing emotional and informational support [21]. In this regard, Ghasemi et al. (2018) [16] showed that perceived social support can protect against disability in older adults. Furthermore, Feng et al. (2014) [22] found that social support plays a mediating role in the relationship between disability and psychological distress in older adults. Additionally, physical disability in older adults has been linked to various issues that make them more vulnerable to psychological and social stressors [6]. Hence, identifying effective stress control strategies significantly promotes optimal health outcomes and healthy aging among older adults. Social support promotes adaptive coping strategies and facilitates the successful adjustment to age-related changes and challenges [23, 24].

**Fig. 1:**
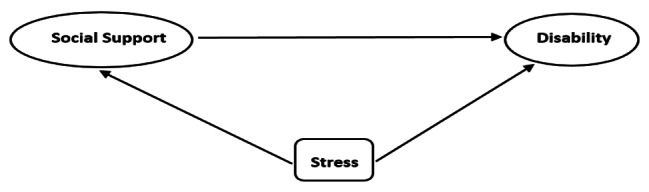
Conceptual model of the present study

In the past two decades, Iranian older population has grown significantly from 1.7% to over 3% [25]. Therefore, the number of older adults with disabilities is likely to increase rapidly over the coming decades [16, 26]. Given that, the prevention of the consequences of disability is vital and limited studies have assessed the disability and its related factors in Iran, this study aimed to determine the relationship between perceived social support and disability with the mediating role of perceived stress (Fig. 1) among older adults residing in Qazvin City, Iran.

## Methods

A descriptive cross-sectional study was carried out between 21st January to 23rd Jun 2022, using a cluster sampling method. For this purpose, the city divided into five zones, including north, south, east, west, and central and one mosque and one park were randomly selected from each zone, and the eligible older adults were chosen from within each of the selected public places. In addition, the samples were recruited from two older adult day care centers that have members from all zones. Inclusion criteria were being 60 years or older, interested in participating in the study, and able to understand and respond to the study questions. Exclusion criteria were having a known psychological illness, severe hearing and physical illnesses that inhibit effective communication.

Questionnaires were completed through face-to-face interviews by the trained research assistants. Participants were informed of their right to withdraw from the study at any time and assured them that their information would be kept confidential and handled under professional ethics guidelines.

The sample size was calculated using G*Power (3.1.0) software. Assuming, the type I error of 0.05, the statistical power of 0.80, and an effect size of 0.5 (f2), a medium effect size, the sufficient sample size was determined at 300.

A demographic information checklist, Multidimensional Scale of Perceived Social Support (MSPSS), World Health Organization Disability Assessment Schedule (WHODAS 2), and Cohen Perceived Stress Scale (PSS-14) were used for collecting the data.

Demographic information checklist includes age, gender, number of children, marital status, education level, living arrangement, job, financial status, and chronic disease history.

### MSPSS questionnaire

The perceived social support was assessed using the MSPSS developed by Zimet et al. in 1988 [27]. This scale is a widely used instrument for measuring perceived social support from family (i.e., My family really tries to help me), friends (i.e., I can count on my friends when things go wrong), and significant others (i.e., there is a particular person with whom I). The MSPSS comprises of three dimensions and 12 items [28]. Participants rated the questionnaire items using a 7-point Likert scale, ranging from 1 (strongly disagree) to 7 (strongly agree). Higher scores indicate more significant levels of perceived social support. Besharat et al. confirmed the validity and reliability of this questionnaire in the Iranian population, reporting a Cronbach’s alpha coefficient of 0.91 for the questionnaire [29].

### WHODAS2 questionnaire

The WHODAS is a standardized questionnaire developed by the World Health Organization (WHO) in 1998 [30]. The WHODAS is a reliable and validated instrument for evaluating disability. The questionnaire consisted of 36 items, rated on a 5-point Likert scale ranging from 1 to 5. The questions were divided into six domains, including understanding and communication, getting around, self-care, getting along with others, life activities, work activities, and participation in social and family activities. The WHODAS questionnaire was used to measure disability severity, with a lower score indicating a higher level of disability [31, 32]. A score of 76 to 100 indicates very severe disability, 51 to 75 severe disability, 26 to 50 moderate disability, 5 to 25 mild disability, and 0 to 4 no disability. Adib-Hajbaghery et al. (2007) confirmed the validity and reliability of the WHODAS questionnaire on older adults in Kashan, Iran [26].

### PSS-14

Cohen et al. developed the PSS-14 in 1983 to measure perceived stress. The PSS has three versions with 4, 10, and 14 items that measure individuals’ perceived stress levels. The PSS has been translated and validated into several languages. In the present study, the 14-item version of the PSS was used to assess perceived stress levels over the past month. The questions are rated on a 5-point Likert scale ranging from never to very often, with scores ranging from 0 to 4. The lowest possible score is 0, indicating no perceived stress and the highest score is 56, indicating a high level of perceived stress [33]. Asghari et al. (2009) confirmed the validity and reliability of this questionnaire among Iranian older adults and reported its Cronbach’s alpha coefficient of 0.84 [34]. Alimohammadi et al. (2019) [35] calculated the internal consistency of this scale at 0.83. Additionally, Rahimi et al. (2023) [36] reported a Cronbach’s alpha coefficient of 0.78 for positive stress and 0.72 for negative stress.

### Statistical analysis

Data analysis was conducted using the Statistical Package for Social Sciences, version 23.0 (SPSS Inc., Chicago, IL, USA) and Mplus 7.4. Quantitative variables were described using means and standard deviations (SD) and qualitative variables by frequencies and percentages. Structural equation modeling (SEM) was employed to determine the mediating role of perceived stress in the relationship between perceived social support and disability. Statistical significance was set at *p* < 0.05.

In SEM, the chi-square test is commonly used as a measure of the model fit. A small and preferably non-significant Chi-square value indicated a good model fit. For the indices Root Mean Square Error of Approximation (RMSEA) and Standardized Root Mean Square Residual (SRMR), values between 0.05 and 0.08 are acceptable, for the Goodness of Fit Index (GFI), values between 0.90 and 0.95, and for the Normed Fit Index (NFI), a value of 0.90 or higher are considered good.

## Results

The study sample consisted of older adults, with a mean age of 68.71 ± 6.13 years, ranging from 60 to 85 years old. The majority of older participants were female (*n* = 160, 53.3%) and married (*n* = 259, 86.0%). The demographic characteristics of the older participants are reported in Table 1.

**Table 1:** Demographic characteristics of the older adults (*n* = 300)

Variable		*N*	Percentage
Gender	Male	140	46.7
	Female	160	53.3
Marital Status	Married	259	86.3
	Single	41	13.7
Educational level	Illiterate	28	9.3
	Writing and reading	80	26.7
	Primary	71	23.7
	Secondary and diploma	56	18.7
	Academic	65	21.7
Living arrangement	With spouse	178	59.3
	With spouse and children	73	24.3
	With children	13	4.3
	With others	17	5.7
	Alone	19	6.3
Job	Unemployed	19	6.3
	Retired	61	20.3
	Employed	119	39.7
	Housewife	101	33.7
Financial status	Low	19	6.3
	Middle	160	53.3
	High	109	36.3
	Excellent	12	4.0
Children	0	25	8.3
	1–2	111	37.0
	3–4	119	39.7
	5 ≤	45	15.0
Chronic disease history	Yes	219	73.0
	No	81	27.0

The mean, standard deviation, minimum, and maximum of the variables under study are presented in Table 2. Based on the results of this study, 9.7% (*n* = 29) of older people did not report disability and the majority of them reported mild (*n* = 146, 48.7%) to moderate (*n* = 90, 30.0%) levels of disability. The rest had sever (*n* = 34, 11.3%) or very sever (*n* = 1, 0.3%) disability.

**Table 2:** The descriptive data of quantitative variables (*n* = 300)

	Variables	Mean	SD	Min	Max
Perceived social support	Family	23.50	4.60	4	28
	Friends	19.06	6.13	4	28
	Other	22.35	5.25	4	28
	Total	64.91	13.10	12	84
Disability	Communicating	12.75	5.04	6	30
	Getting around	9.84	4.97	5	25
	Self-care	5.78	2.62	4	17
	Getting along with people	8.78	2.62	5	25
	Life activities	7.56	3.83	4	20
	Work task	9.91	4.64	5	25
	Participation in society	18.08	7.11	8	36
	Total	72.70	24.88	37	146
Perceived Stress	Total	37.86	8.66	15	64

**Fig. 2:**
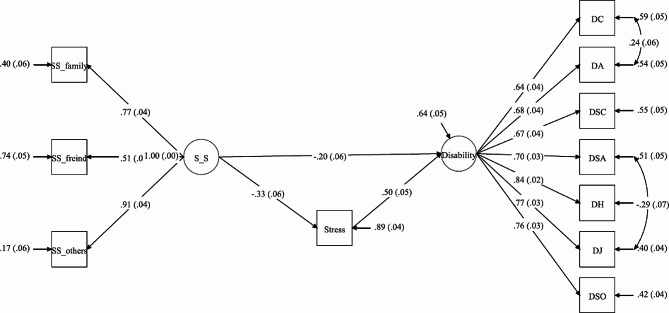
The results of the structural model of the research. Note: SS = Social Support, SS_family = Social Support_Family, SS_friends = Social Support_Friends, SS_others = Social Support_Others, DC = Communicating, DA = Getting around, DSC = Self-care, DSA = Getting along with people, DH = Life activities, DJ = Work task, DSO = Participation in society

The present study employed a SEM approach to examine the potential mediating effect of perceived stress on the relationship between perceived social support and disability. The results of the SEM indicated a significant association between perceived social support and disability [β (SE): -0.20(0.06), *p* < 0.001] and between perceived stress and disability [β(SE):0.50 (0.05), *p* < 0.001]. Further analyses revealed that perceived stress mediated the relationship between perceived social support and disability, as evidenced by a significant indirect effect [β(SE): -0.17(0.03), *p* < 0.001] (Table 3). Moreover, the results indicated that 20% of the variance of disability was explained by perceived social support (*p* < 0.001). The factor loading of the studied constructs is depicted in Table 4. Table 3; Fig. 2 present the model’s fit indices.

**Table 3:** Factor loadings of the studied constructs in the SEM analysis

Factors	Estimate(S.E.)	
	Unstandardized	Standardized
**Disability**		
Communicating	1.00(0.00)	0.64(0.04)
Getting around	1.05(0.09)	0.68(0.03)
Self-care	1.04(0.10)	0.67(0.03)
Getting along with people	1.09(0.11)	0.70(0.03)
Life activities	1.30(0.11)	0.84(0.02)
Work task	1.20(0.11)	0.77(0.03)
Participation in society	1.18(0.11)	0.76(0.03)
Perceived Social support		
Family support	1.00(0.00)	0.77(0.04)
Friends	0.65(0.08)	0.50(0.05)
Other	1.17(0.11)	0.91(0.04)
	**Disability**	
Perceived Social support	-0.16(0.05)	-0.20(0.06)
	**Stress**	
Perceived Social support	-0.43(0.08)	-0.33(0.06)
	**Disability**	
**Stress**	0.32(0.04)	0.50(0.05)

**Table 4:** The results of total, direct, and indirect relationships of perceived social support and disability and the role of perceived stress as a mediating variable in the SEM model

Variables	Model	
Relationship between social support and disability	Regression Coefficients (Standard Errors)	
	Standard	Non-standard
Total	-0.36(0.06) *P* < 0.001	-0.30(0.06) *P* < 0.001
Direct pathway	-0.20(0.06) *P* = 0.001	− 0.16(0.05) *P* = 0.001
Indirect	-0.17(0.03) *P* < 0.001	-0.14(0.03) *P* < 0.001
Goodness of fit		
χ2 (df)	76.39 [40]	
RMSEA	0.05(0.04, 0.07)	
CFI/ TLI	0.97/0.96	
SRMR	0.03	

χ2 = goodness-of-fit chi-square; CFI = Comparative Fit Index; TLI = Tucker-Lewis Index; RMSEA = root mean square error of approximation; SRMR = standardized root mean square residual, within group

## Discussion

The current study examined the potential mediating effect of perceived stress on the relationship between perceived social support and disability among older adults residing in Qazvin, Iran. The majority of the study sample reported mild to moderate levels of disability. Previous national studies have reported different results. For instance, Mozafari et al. (2016) showed that most older adults experience low to moderate levels of disability [37]. However, Baghery Kakhki et al. (2020) found that older adults have very low levels of disability [25]. Additionally, Jafari et al. (2018) reported that most older adults experienced moderate to severe disability [38]. Noei et al. (2017) also found a significant increase in the level of disability among retired older adults with advancing age [39]. The inconsistencies in the results of previous studies most likely due to differences in data collection techniques, variations in the environment, and discrepancies in the mean age of the older adults being studied.

The results of the current study indicated that the lowest levels of disability were in self-care dimension. Additionally, family members were the primary source of perceived social support for these older adults. Baghery Kakhki et al. (2020) and Adib-Hajbaghery et al. (2009) reported that self-care had the lowest level of disability among older adults [25, 26]. Similarly, Noei et al. (2017) found that self-care had the lowest levels of disability among older men [39]. The lower levels of self-care disability among older adults may be attributed to the support they receive from family members for performing daily activities.

The present study consistent with the results of Huang et al. (2020) identified a significant negative correlation between perceived social support and disability among older adults [40]. Feng et al. (2014) demonstrated that an increase in perceived social support could reduce functional disability among individuals aged 75 years and higher [22]. Furthermore, Ghasemi et al. (2022) reported that social support is a protective factor against disability among older adults [16]. Social support plays a crucial role in boosting an individual’s ability to cope with stress and become more resilient [40]. Also, having support from friends and peers is vital to protect against feelings of hopelessness and anxiety [41]. So, individuals with greater social support experience better physical and mental health, as well as improved quality of life, which can reduce disability [17, 18].

The present study provided evidence for the mediating role of perceived stress in the relationship between perceived social support and disability among older adults. Specifically, an increase in perceived social support leads to reduced perceived stress and, in turn, a decrease in disability among older adults. Social support can have a positive impact on psychological well-being by promoting positive thinking and healthy behaviors during stressful situations [42]. Perceived social support can enhance an individual’s ability to manage stress and difficult situations, ultimately leading to a better quality of life [16, 43]. The buffer-stress hypothesis suggests that social support can protect individuals from stressors by enhancing their coping abilities. This can help them reduce physical and psychological symptoms and chronic conditions during times of severe stress [19, 21]. So, perceived social support can reduce functional limitations and disability by prevention of chronic illnesses and promoting health of older people [16].

## Conclusion

The results of this study emphasize for strengthen social support as a preventive factor for stress-related problems among the older population. So, policymakers need to allocate more resources to design of educational and counseling programs for promoting social support to reduce the disability rate among older adults.

### Limitations

Limitations of the present study included using self-report measures to fill out questionnaires, which may have resulted in some seniors not providing genuine answers. However, efforts were made to minimize this limitation by explaining the study objectives. Another limitation was the focus on community-dwelling older adults, which may limit the generalizability of the results to institutionalized older adults.

### Implications for practice

The reflection of the results of this research on health officials and policymakers will lead to more comprehensive and effective planning for the rehabilitation and support of older adults and their families, as well as the prevention of chronic disease-related disabilities. Furthermore, the results of the current study could serve as a motivation for investigating disability at a broader provincial and national level.

### Suggestions for future studies

It is suggested to conduct further studies to investigate the prevalence of disability and its related factors among institutionalized older adults and those residing in other cities in Iran. Additionally, it is suggested that an intervention study be conducted on the impact of social support on perceived stress and disability.

## Data Availability

The data set used in the present study will be available from the corresponding author on reasonable request.

## Abbreviations

MSPSS: Multidimensional Scale of Perceived Social Support
WHODAS 2: World Health Organization Disability Assessment Schedule
PSS-14: Cohen Perceived Stress Scale
ICF: International Classification of Functioning, Disability, and Health
